# Clinical Society

**Published:** 1897-11-06

**Authors:** 


### CLINICAL SOCIETY.

### One-sided Blushing on Eating.

At the last meeting of the Clinical Society a curious case was shown by Dr. Parkes Weber. It was that of a young man who exhibited a localised blush on the left cheek when eating. There was sweating also over part of the corresponding area, but the sweating did not extend to the ear which was involved in the blush. Mere movement of the jaw did not produce the phenomena, which, however, were set up by placing vinegar in the mouth even though the jaws were kept still.

The patient had formerly suffered from a suppurating gland behind the ear, and it was supposed that some of the vaso-constrictor branches of the cervical sympathetic were involved in the scar and were irritated when the gland became distended.

